# Efficiency of Silver Impregnated Porous Pot (SIPP) Filters for Production of Clean Potable Water

**DOI:** 10.3390/ijerph9093014

**Published:** 2012-08-24

**Authors:** Oranso Mahlangu, Bhekie Mamba, Maggie Momba

**Affiliations:** 1 Department of Chemical Technology, University of Johannesburg, P.O. Box 17011, Doornfontein 2028, South Africa; Email: mahlanguoranso@yahoo.com; 2 Department of Environmental, Water and Earth Sciences, Tshwane University of Technology, 175 Nelson Mandela Drive, Pretoria 0002, South Africa; Email: mombamnb@tut.ac.za

**Keywords:** silver impregnated porous pot filter, chlorophyll *a*, total organic carbon, flow rate, turbidity

## Abstract

The Silver Impregnated Porous Pot (SIPP) filter is a product of the Tshwane University of Technology manufactured for the production of safe drinking water at a household (home) level. Two SIPP devices were assessed for the reduction efficiency of chemical contaminants such as calcium, magnesium, iron, arsenic, fluorides and total organic carbon (TOC) as well as microbial contaminants from environmental samples. Turbidity change after filtration, together with correlation between chlorophyll *a* in the feed water and SIPP’s flow rates were also evaluated in order to give comprehensive guidelines on the quality of intake water that could be filtered through the filter without causing a significant decrease in flow rate. The SIPP filters removed contaminants from environmental water samples as follows: 70% to 92% iron, 36% to 68% calcium, 42% to 82% arsenic, 39% to 98% magnesium, 39% to 95% fluorides, 12% to 35% TOC and 45% to 82% turbidity. The SIPP filters had initial flow rates of 1 L/h to 4 L/h but the flow rates dropped to 0.5 L/h with an increase in cumulative volume of intake water as the filter was used. Turbidity and chemical contaminant reduction rates decreased with accumulating volume of intake water but the filter removed Ca, Fe and Mg to levels that comply with the South African National Standards (SANS 241) and the World Health Organization (WHO) guideline values. However, the SIPP filters cannot produce enough water to satisfy the daily drinking water requirement of a typical household (25 L/p·d). Chlorophyll *a* was associated with a decrease in the flow rate through the SIPP filters.

## 1. Introduction

Provision of adequate clean potable water to a nation is a complex challenge that calls for innovation. There is a lack of clean drinkable water supplies in many communities of Third World countries. The shortage of clean potable water has become a major concern worldwide. Industrialisation is a major cause of contamination of groundwater and surface-water sources [1].Some of these water sources contain dissolved ions, oils and organics which may exceed SANS and WHO guideline values. Clay minerals are one of the adsorbents that could possibly remove these contaminants [2]. The porous clay pot household filter is a device that uses an adsorption mechanism to remove chemical contaminants from contaminated water. The most common locally-made clay pot filter in South Africa is the Potters for Peace (PFP) clay pot originally developed by the Central American Research Institute of Industrial Technology (ICAITI) in the 1980s. It has a capacity of 6 L to 8 L and is manufactured from natural materials [3] such as clay and fine sawdust that are mixed in an appropriate mixing ratio based on availability of local materials. The PFP filter can be easily constructed manually but usually the PFP clay-pot filter is constructed by pressing the clay mixture in a mould using a hand-operated hydraulic truck jack, and then firing at 860 °C. The sawdust is burnt off during firing leaving tiny pores that block off impurities while letting water seep through. Colloidal silver, known for its antimicrobial properties, is normally incorporated in these filters in the form of silver nitrate or a colloidal silver solution. It is assumed that colloidal silver reduces bacterial contamination as it kills bacteria by inactivating their metabolic enzymes or by attaching to the cellular membrane [4]. Clay filters such as the PFP filter, have filter pore sizes of 0.1 µm to 10 µm and have flow rates of between 1 L/h and 3 L/h [4]. ICAITI [3] noted that the flow rate declines with use and the decline rate could be up to 64% depending on the turbidity of the intake water and the flow rates may decline to as low as 0.5 L/h in some circumstances [4]. Ceramic filters remove contaminants mostly by size exclusion and a large proportion of silts and solids can be removed by washing. The PFP clay pot can reduce turbidity by between 30% and 100% as reported by Lantagne [5]. Ceramic filters may have a lifespan of up to seven years and still remove microbial and chemical contaminants from water [5]. Ceramic filters have the advantage of producing quality water with good taste and they are easier to maintain than biological sand filters where some of the sand (5 cm layer of the top layer) has to be removed, washed and repacked again or replaced with new sand [5].

Although a lot of research has been done on clay pots, data on the removal of chemical contaminants by these filters are still lacking. In addition, these filters have not been extensively tested with South African surface water and groundwater sources to evaluate whether they produce water of good quality that complies with SANS 241 [6]. Most of the research has been concentrated on the removal of micro-organisms such as viruses, protozoa, bacteria and helminths. Less or no research has been done on the removal of dissolved cations and anions, oils and organics which may exceed guideline values. There are various small-scale devices that have been designed for the removal of arsenic from contaminated water. The technologies have been applied in water treatment. The filters are believed to remove arsenic from feed water through processes such as adsorption, and surface precipitation [7]. Ceramic filters (made from locally available material) have been shown to remove arsenic through biological oxidation of iron (II) [8].

The selection of the clay pot for evaluation of chemical contaminant removal from South African water sources was based on several selection criteria which include ease of construction, maintenance, use and cost-effectiveness. The objective was to evaluate the removal efficiency of the Silver Impregnated Porous Pot (SIPP) filter for removing chemical contaminants. Specific objectives were to:

- Evaluate the removal efficiency for calcium (Ca^2+^), iron (Fe^3+^), magnesium (Mg^2+^), arsenic (As^3+^), total organic carbon (TOC) and fluorides (F^−^) from contaminated water.
- Determine the flow rate of the filter and compare with results given in the literature.
- Determine whether the filter removes suspended particles (turbidity) from contaminated water.
- Evaluate the effect of turbidity on the flow rate of the filter.
- Determine the relationship between chlorophyll *a* in the feed water and noted flow rate of the SIPP filter.

## 2. Materials and Methods

### 2.1. Filter Description

**Figure 1:**
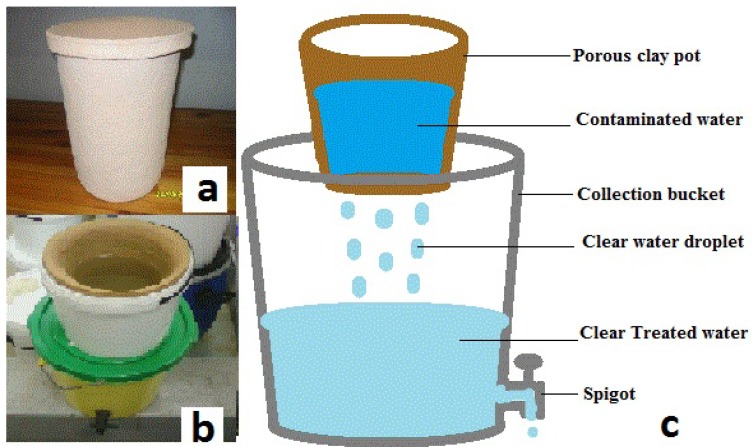
Illustration of the SIPP Filter: (**a**) the silver impregnated clay pot that serves as the filtering unit; (**b**) the clay pot fitted into a 10 L bucket (receptacle) and placed on top of a 25 L collection vessel; (**c**) schematic diagram of a complete SIPP filter.

Ten SIPP filters were constructed. For the purpose of this study, two SIPP filters were evaluated. The remaining eight filters were retained to evaluate social acceptance of the SIPP filters focusing on homes in the rural areas in South Africa. Results for social acceptance will be presented in our future publications. The SIPP filters with capacities of 5 L to 6 L were constructed in the laboratory (Tshwane University of Technology) and are henceforth called the Silver Incorporated Porous Pot (SIPP) filters. The study was commissioned and funded by the Water Research Commission of South Africa (WRC Project No. K8/810) and the construction of the filters was in fulfilment of the objectives of the project. Brown clay was used in its construction and the clay was impregnated with silver nano-particles by mixing the clay with silver nitrate, saw dust and paper fibre to make a dough. The dough was shaped into a flower pot, dried and fired at 887 °C for 8 h to 9 h to remove the combustible material. Figure 1 shows the complete SIPP filter assembly. The filtering unit was the porous clay pot. Filtered water is collected in a clean collection bucket with a capacity of 25 L. The bucket is fitted with a tap to draw the treated water in order to reduce recontamination of water when it is drawn for use.

### 2.2. Baseline Determination of Flow Rate and Filtration of Spiked Samples

Prior to assessing the filters separately for performance with environmental water samples, deionised water was filtered through and the flow rates were measured volumetrically. An average of the flow rates (which remained similar) was calculated. A total volume of 30 L (six filtration times with 5 L per trial) was passed through each filter before spiking experiments. Chemical contaminants of interest were then spiked in the deionised water to obtain desired concentrations of 200 mg/L Ca, 100 mg/L Mg, 5 mg/L Fe, 10 mg/L As and 5 mg/L F. Each contaminant was spiked separately. Pure standards of 1,000 mg/L Ca, Mg, Fe, As and F were used. All filtration experiments with spiked water samples were repeated five times (total filtered water = 125 L). Deionised water spiked with microbial contaminants was also passed through the filters. A total volume of 30 L (six filter runs per filter) was filtered for this purpose.

### 2.3. Sample Collection and Filtration

The SIPP filters were evaluated for their efficiency to reduce calcium, magnesium, iron, arsenic, fluorides and total organic carbon from environmental (surface and ground) water sources with low and high turbidity following *Standard Methods* [9]. These water samples (river water and borehole water) were sourced from three provinces of South Africa (Gauteng, Mpumalanga and North West). The SIPP filters were also evaluated for the removal of microbial contaminants (bacteria, viruses and protozoa) and results have been presented in our previous work [10].

Water samples were collected from the Apies River (surface water with low turbidity), Delmas Municipality Borehole No. 7 (groundwater with low turbidity), Wallmannsthal (groundwater with high turbidity) and Hartbeespoort (surface water with high turbidity). Water sources were classified as ‘low and high turbidity’ when the turbidity reading was below (low turbidity) and above (high turbidity) the SANS 241 [6] recommended guideline value of less than 1 NTU. Turbidity of water sources was measured onsite (Eutech TN-100 turbidimeter). Collected water was stored in 50 L barrels with covers. Four barrels were used at each time of sampling; hence about 200 L was collected per sampling time. It has to be noted that we also evaluated four other filters in the course of assessing the efficiency of the SIPP filters. This is the reason why large volumes of water were collected from each sampling site. Sampling was done six times at each sampling point.

The collected water was filtered through the SIPP (5 L) filters upon arrival in the laboratory (TUT). Filtration was carried out for 3 h and was done so on the assumption that enough water would have been produced for family drinking and cooking needs. Different volumes of filtrates were collected over the 3 h period of filtration at 1 h intervals, in order to evaluate whether there was a difference in chemical contaminant removal efficiency at different times and to make necessary adjustments or recommendations. One sample was collected for analysis at hourly intervals after the start of the filter run from each filter. Each sample was analysed three times for the analyte of interest. An average (± standard deviation) was calculated from the combined results of the two filters. In cases where the pre-filtered water was found to have lower contaminant concentrations that fell within the recommended guideline values by SANS 241 [6], the water was spiked with the chemical of interest. Appropriate concentrations of contaminants of interest were spiked accordingly to adjust final concentrations in line with concentrations described before for synthetic water samples. The amount of contaminant added was determined by the initial concentration of the analyte in the feed water. Spiking of samples was mainly done to evaluate whether the filters would reduce the concentrations of the contaminants of interest to allowable levels recommended by SANS 241 [6].

### 2.4. Flow Rate and Turbidity Determination

Flow rates were determined by measuring the volume of water (L) collected for each filter per unit time (h). Deionised water (2 µS/cm to 3 µS/cm) with low turbidity and contaminant concentration was filtered first to establish a benchmark for the filters’ flow rates as mentioned before. It was measured at a later stage when environmental water samples with lower and higher turbidity levels were filtered. Turbidity determination was useful in accomplishing two objectives. Firstly, the study was aimed at evaluating the filters’ turbidity removal efficiency. Secondly, we needed to scale up the effect of turbidity on the flow rates of the filters. Turbidity in nephelometric turbidity units (NTU), for all samples was determined using a Eutech TN-100 turbidimeter calibrated with 0.2 NTU to 800 NTU calibration standards.

### 2.5. Determination of the Contaminant Removal Efficiency of the SIPP Filters

Chemical analysis of calcium, magnesium, iron and arsenic were performed by atomic absorption spectrophotometry (AAS) using a VARIAN 220 FS model equipped with an air acetylene flame and nitrous oxide as a support gas for arsenic and calcium analysis [9]. Standard solutions of the pure metal ions (1,000 mg/L) were obtained from the chemical store of the Tshwane University of Technology. Working standards (200 mg/L, 100 mg/L, 10 mg/L and 5 mg/L for calcium, magnesium, iron and arsenic, respectively) were prepared from the 1,000 mg/L bulk standards. Deionised water was used in all experimental measurements. Accuracy of the Varian 220 FS was validated using a graphite furnace AAS (Perkin Elmer, Analyst 600 model).

### 2.6. Fluoride Analysis

A Metrohm 713 pH meter was used together with a fluoride electrode for all experimental measurements of fluorides from surface water and groundwater samples with low and high turbidity. A silver/silver chloride electrode (Ag/AgCl 6.0228.00) was used as the reference electrode. Fluoride standard solutions (0.1 mg/L to 100 mg/L) were prepared using analytical reagent (AR) grade sodium fluoride (NaF) and a calibration curve was drawn as log concentration *versus* potential difference (mV). Fluoride samples and fluoride standard solutions were diluted with TISAB II (total ionic strength adjuster buffer) in a 1:1 ratio. This buffer helps in reducing the variation of the ionic strength in standards and samples. TISAB II was obtained from the Tshwane University of Technology chemical store. The buffer contains a product which forms a complex with all the ions that could result in interferences when determining fluorides and it de-complexes all complexed fluorides into free detectable fluoride ions. The ions (F^-^) can then be detected with the ion-selective electrode [11].

### 2.7. Analysis of Total Organic Carbon (TOC)

Total organic carbon (TOC) concentrations in all samples (filtered and unfiltered) were determined using a TOC combustion analyser (TEKMAR DOHRMANN APOLLO 9000 model). A calibration curve was plotted using results of 1.0 mg/L, 5.0 mg/L, 10.0 mg/L, 20.0 mg/L and 25.0 mg/L potassium hydrogen phthalate standards. Vials with penetrable Teflon septum were filled with 40 mL of samples to be analysed and put into the TOC analyser auto-sampler rack. Quadruplet readings deduced from the calibration curve were recorded for each sample.

### 2.8. Chlorophyll *a* Analysis

The main aim in the determination of chlorophyll *a* was to quantify the amount of algae in unfiltered water samples and to correlate this with the filters’ flow rates. Briefly, our aim was to establish how higher concentrations of algae (measured as chlorophyll *a*) were associated with the observed flow rates of the filters. Chlorophyll *a* concentrations were determined using *Standard Methods* [9]. A CENTRO 8 centrifuge was used to centrifuge all samples at 3,600 r/min for 5 min and an ultrasonic cell disruptor (VIRSONIC 100 model) was used to disrupt algal cells in order to extract the chlorophyll *a.* Optical density was measured using a spectrophotometer (SPEKOL 1300 model) at 750 nm and 664 nm prior to acidification and at 750 nm and 665 nm after acidification of samples with 0.1 mL of 0.1 M hydrochloric acid [9].

### 2.9. Statistical Analysis

The study also focused on carrying out statistical analysis to evaluate the performance of the filters on the reduction of different contaminants from surface water and groundwater samples with low and high turbidity, at the 95% confidence interval. The Stata V10 statistical package (StataCorp LP, College Station, TX, USA) was used to evaluate whether there was a significant difference in the reduction of chemical contaminants by the SIPP filters at the different times of filter run. Also investigated were the following parameters: the correlation between chlorophyll *a* concentration and flow rates of the SIPP filters; the correlation between turbidity of intake water and flow rates of the SIPP filters; and the correlation between chlorophyll *a* concentration and turbidity of intake water.

### 2.10. Investigation of Cost and Maintenance of the Filters

Determination of the cost of production and maintenance costs of the filters was done following similar methods reported in our previous work on other cost-effective household devices [12,13]. This step was undertaken to compare construction, water production and maintenance costs of each SIPP filter with other filters, such as ceramic candle filters, which are available on the market. 

## 3. Results and Discussion

### 3.1. Flow Rates and Turbidity Reductions

As stated earlier on, average results of the two filters are presented in this manuscript. The flow rates of the SIPP filters were observed to be highest in the first hour of filter run and lowest in the third hour of filtration. The flow rates were observed to decrease with continuous use of the SIPP filters. The SIPP filters had high flow rates on filtration of spiked deionised water. This could be as a result of lower turbidity levels of the intake water. A fluctuation in the flow rates of the filters was observed; however, the causes of the fluctuations were not quantified. 

**Figure 2:**
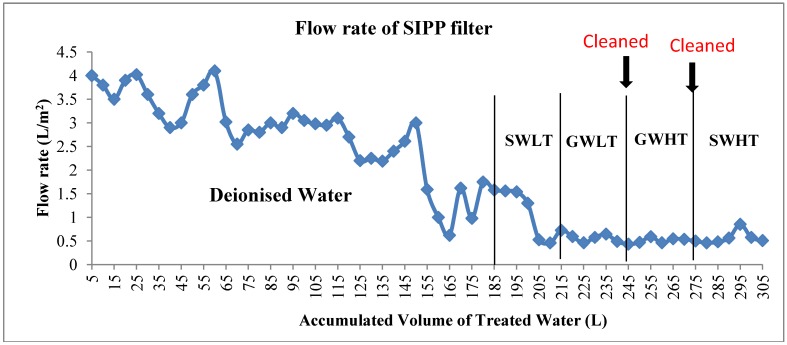
Flow rate (average of two filters) of SIPP filter for different water source samples.

The filters’ flow rates were found to be higher on filtration of surface water with low turbidity (SWLT, Figure 2) compared to filtration of other environmental water source samples. The flow rates declined with an increase in volume of water filtered through. The highest flow rate obtained on filtration of environmental water sources was 1.56 L/h (190 L). It was observed that the flow rates of the SIPP filters were lower at higher turbidity levels and higher at lower turbidity levels of filtered water; however, statistical results indicated a weak negative correlation between the filters’ flow rate and turbidity (*r* = −0.16). 

On filtration of groundwater with low turbidity (GWLT), groundwater with high turbidity (GWHT) and surface water with high turbidity (SWHT), the filters’ flow rates remained constant at about 0.6 L/h. Filter cleaning before filtration of GWHT and SWHT did not improve the filter flow rate. It may be hypothesised that foulants build up not only on the filter surface, but become trapped within the filter pores, causing clogging. These trapped foulants are difficult to remove during filter cleaning, thereby impeding proper flow through the filter. The flow rates declined to very low levels that were not suitable for the production of suitable volumes of product water for a larger family. Statistically, there was a significant difference in the flow rates between spiked water samples and environmental water samples (*p* ≤ 0.05). Flow rates on filtration of environmental water samples remained the same (*p* ≥ 0.05).

Higher turbidity measurements were obtained on filtration of SWHT. Before filtration the highest recorded turbidity was 42.9 NTU and the lowest turbidity recorded was 1.47 NTU. The filters removed turbidity to allowable levels from all water sources. The SIPP filters were more effective in turbidity removal when the turbidity of the intake water was greater than 10 NTU. Filter washing resulted in an improvement in turbidity reduction by the SIPP filters. Lantagne [5] reported turbidity reduction efficiencies of 30% to 100%, which is in agreement with our results, where the reductions were within the ranges of 44.9% to 93.9% (SWLT), 40.1% to 60.7% (GWLT), 35.9% to 55.1% (GWHT) and 78.0% to 87.0% (SWHT). Our results also showed that filtration of high-turbidity water reduced the flow rates of the filters, concurring with the findings reported in the literature [4]. There was a significant difference in the hourly reduction of turbidity by the SIPP filters (*p* ≤ 0.05). Turbidity reduction by the filters is believed to be enhanced by clogging of the filters’ pores. However, at times it was noted that the turbidity of the filtrate in the third hour was higher than the turbidity of the filtrate in the first and second hour of filter run. It is hypothesised that particles that pass through the filter collect and settle on the surface of the spigot of the collection vessel. These particles increase in concentration and are washed into the filtrate resulting in low turbidity reduction in the third hour of filter run. Turbidity reduction by the filters on all environmental samples was the same (*p* ≥ 0.05).

### 3.2. Reduction of Chemical Contaminants

#### 3.2.1. Removal of Chemical Contaminants from Spiked Water Samples

Table 1 presents preliminary results of spiking deionised water with the contaminants of interest. Thirty samples were analysed for each contaminant. Results showed removal efficiencies of greater than 50% for all contaminants, with the exception of fluorides. Calcium, magnesium and iron were significantly removed to levels below the recommended guideline values. However, the SIPP filters did not remove arsenic and fluorides to levels below the SANS 241 and WHO guideline values. The filters removed all chemical contaminants, except for arsenic and fluorides, at each hour of filter run to the same extent (*p* ≥ 0.05). The filters showed significant variation in the hourly arsenic and fluoride removal efficiencies (*p* ≤ 0.05).

The flow rates of the filters were found to be higher on filtration of synthetic water samples than when environmental water samples were filtered. There was, however, no determinable relationship between the reduction in chemical contaminants and flow rates as some contaminants were removed better at similar flow rates while others were not. For the spiked samples, each chemical contaminant was spiked after five trials had been run for the pollutant analysed. It may be possible that the filtration of previous samples affected the removal of contaminants from samples in subsequent filter runs. The filters were not cleaned before filtration of the succeeding feed water samples with different analytes which were spiked separately one at a time. In this study, two parameters were used to determine whether the capacity of the filter had been exhausted and it needed cleaning; the first parameter being a significant decline in the flow rates, and the second a decline in the filters’ ability to reduce turbidity. The filters were therefore not cleaned between filter runs when the flow rates were still high. 

**Table 1:** Chemical profile of spiked water samples for preliminary analysis.

Contaminant of interest	Guidelines (mg/L)		Spiked levels (mg/L)	Removal (%)	No. of samples
	SANS 241	WHO			
Calcium	150–300	100–300	200	56.3 (±11.1)	30
Magnesium	70–100	200	100	66.7 (±6.7)	30
Iron	0.2–2.0	1–3	5	88.7 (±12.4)	30
Arsenic	0.0005	0.0002	10	53.9 (±20.4)	30
Fluoride	1.0–1.5	1.5	5	43.5 (±31.5)	30

#### 3.2.2. Removal of Chemical Contaminants from Environmental Water Samples

In this study, 120 L of contaminated environmental water was filtered and the levels of contaminants (average ± standard deviation) before treatment for each water source are presented in Table 2. SANS 241 and WHO guideline values for drinking water [6,14] are presented in Table 1. Water from each water source was filtered six times through each filter and one sample was taken from each filter at hourly intervals during each filter run. Each sample was analysed three times. The first 30 L of contaminated water comprised the surface water sample with low turbidity (SWLT) (185 L to 215 L), the second 30 L comprised the groundwater sample with low turbidity (GWLT) (220 L to 245 L), the third 30 L comprised the groundwater sample with high turbidity (GWHT) (250 L to 275 L) and the last 30 L comprised the surface water sample with high turbidity (SWHT) (280 L to 305 L). The samples were assayed for all the chemical contaminants. There was a variation in the distribution of chemical contaminants in the water sources. It can be seen from Table 2 that the concentrations of some contaminants in environmental water sources were generally lower than the SANS 241 and WHO guideline values. The contaminants, with the exception of As and TOC, were therefore spiked to desired concentrations as mentioned before. Turbidity levels of GWLT differed from turbidity levels of GWHT and SWHT (*p* ≤ 0.05) but were similar to turbidity levels of SWLT (*p* = 0.99). Similar turbidity levels between GWHT and SWHT were noted (*p* = 0.75).

Table 3 shows the overall performance of the SIPP filters in removing contaminants from different source water samples. The results are averages (±standard deviations) of six trials conducted per sampling site for the two filters used. A total of 36 samples were analysed for each parameter.

**Table 2:** Physicochemical profile of water sources before treatment (mean ± standard deviation) together with SANS 421 [6] and the World Health Organization guideline values [14] for drinking water.

Water Type	Turbidity (NTU)	Analyte Concentration (mg/L)					
		Ca	Mg	Fe	As	F	TOC
SWLT	11.9 (±10.2)	137.1 (±48.7)	65.9 (±42.9)	1.03 (±0.5)	4.90 (±1.2)	3.39 (±1.7)	7.71 (±0.5)
GWLT	2.17 (±0.8)	158.5 (±47.2)	58.5 (±35.9)	0.20 (±0.2)	9.48 (±2.9)	7.72 (±11.1)	7.12 (±1.0)
GWHT	8.39 (±5.4)	23.9 (±9.6)	59.6 (±38.9)	0.23 (±0.0)	8.12 (±1.5)	0.49 (±0.1)	5.87 (±0.8)
SWHT	40.4 (±4.1)	14.9 (±2.67)	25.6 (±1.1)	0.29 (±0.0)	5.03 (±0.9)	0.85 (±0.1)	4.81 (±0.7)

**Table 3:** Average reduction of contaminants by SIPP filter from different water sources.

Water type	Analyte Reduction (%) (average ± standard deviation)						
	Turbidity	Ca	Mg	Fe	As	F	TOC
SWLT	69.4 (±24.5)	43.4 (±19.2)	49.5 (±19.6)	70.0 (±8.1)	57.3 (±18.8)	39.6 (±13.4)	15.8 (±3.0)
GWLT	50.4 (±10.3)	67.5 (±6.8)	39.8 (±13.7)	92.5 (±2.6)	42.6 (±14.6)	42.9 (±19.5)	12.3 (±3.1)
GWHT	45.5 (±9.6)	36.5 (±17.9)	71.5 (±7.9)	87.8 (±4.6)	52.6 (±19.1)	56.9 (±18.5)	18.6 (±6.6)
SWHT	82.5 (±4.5)	68.8 (±11.8)	98.4 (±0.1)	79.1 (±3.3)	82.9 (±7.0)	95.1 (±3.6)	35.2 (±5.3)

##### 3.2.2.1. Filter Performance in Treating SWLT

On filtration of SWLT, the filters removed turbidity, calcium, magnesium and iron to levels below the recommended guideline values. The removal of these contaminants was significant. Removal of these contaminants was highest when their initial concentrations in the feed water were higher. Arsenic and fluorides were removed to a lesser extent by the filters on treating SWLT. Although higher reductions were often observed, these contaminants were not removed to levels below the SANS 241 and WHO guideline values. There were very low observable reductions of TOC. Arsenic and TOC were greatly reduced at lower initial concentrations in the feed water. The filters were not able to achieve removal efficiencies of 50%. Higher reductions of the contaminants were associated with lower flow rates. This could be as a result of increased contact time between the filters and contaminated water. A high reduction of contaminants was observed when the turbidity of intake water was higher. This could be due to the fact that with higher initial contaminant concentrations in the feed water, the turbidity was also higher. Initial concentration of the contaminants in feed water was found to be associated with the removal of the contaminant. Generally, removal of all contaminants (with the exception of arsenic and TOC) was found to improve when their initial concentration in the feed was higher. Filter cleaning was not performed on filtration of this water type because the filters’ flow rates remained high (about 1.5 L/h) for the first four trials conducted. In addition, contaminant removal efficiencies were still high (with the exception of arsenic, fluorides and TOC). Statistical results indicated that there was no significant difference in reduction of contaminants by the SIPP filters after 1 h, 2 h and 3 h (*p* = 0.82) on filtration of SWLT.

##### 3.2.2.2. Filter Performance in Treating GWLT

There was lower observable turbidity reduction by the SIPP filters on filtration of GWLT. This could be due to the lower initial turbidity. A 60% reduction was observed and turbidity levels in the filtrate were within the recommended guideline values by SANS 241 and WHO [6,14]. There was also greater removal of calcium, iron and fluorides than was the case when filtering SWLT. Calcium and iron were significantly removed while the removal of fluorides fluctuated (*i.e.*, on some occasions the removal was significant). Arsenic, magnesium and TOC were not significantly removed by the filters. Concentrations of these foulants were higher than the recommended guideline values in the filtrate. The flow rates of the filters remained constant at about 0.5 L/h on filtration of GWLT. The reduction of calcium and iron could be associated with the lower flow rates (smaller standard deviations at constant flow rate). The removal of magnesium, arsenic and fluorides, however, could not be associated with the filters’ flow rates. Removal of these contaminants varied (higher standard deviation) at constant flow rates. It may be hypothesised that the observed reduction in the removal of magnesium by the filters could be due to the lower measured turbidity of the intake water. Lower filter performance was also noticeable in arsenic and TOC reductions. It was also determined that the initial analyte concentration in the GWLT feed water exerted an influence on the removal of the contaminants by the filters. Higher removals of contaminants (with the exception of arsenic and TOC) were associated with higher initial analyte concentrations in the feed water. The filters were not cleaned at this stage or during the course of filtration of GWLT. The effect of cleaning on the filters’ performances in treating GWLT could therefore not be quantified. Contaminants were removed equally well by the SIPP filters throughout the 3 h duration of filter run (*p* ≥ 0.05). 

##### 3.2.2.3. Filter Performance in Treating GWHT

Before filtration of GWHT, the filters were cleaned as the flow rates had decreased significantly in comparison to those of the initial stages of filter use. The filter flow rates did not improve after cleaning, but they remained constant at about 0.5 L/h. On average, there were lower observed removals (compared to removals on filtering SWLT and GWLT) of turbidity and calcium by the filters on filtration of GWHT. Reduction of magnesium, fluorides and TOC was improved and higher compared to observed reductions on filtration of previous water sources (SWLT and GWLT). Turbidity and arsenic were not always reduced to levels below the recommended guideline values. The rest of the contaminants (calcium, magnesium, iron and fluorides) were significantly removed to levels below the recommended guideline values. All the contaminants were removed to the same extent at each hour of filter run (*p* ≥ 0.05). This showed that contact time was not a factor on the filters’ performance on removing contaminants from GWHT source water. As mentioned earlier on, the filters’ flow rates remained constant even after cleaning. Therefore no clear conclusion could be drawn on how the flow rates were associated with overall removal of contaminants. Higher reductions of the chemical contaminants were observed when the turbidity of feed water was higher. It may therefore be reasoned that contaminant removal by the SIPP filters was associated with turbidity of filtered water when GWHT samples are filtered. There was an improvement in the reduction of magnesium, arsenic, fluorides and TOC (Table 3), which could be as a result of cleaning the filters before filtration of GWHT. Filter cleaning resulted in a decrease in observed turbidity reduction. This could be as a result of the removal of the particles that clogged the filter pores and reduced the filter pore size. However, this reasoning is not conclusive, as the flow rates remained constant. There might be other factors which affected turbidity reduction by the filter. Higher removals of contaminants (with the exception of arsenic and TOC) were associated with higher initial analyte concentrations in the feed water.

##### 3.2.2.4. Filter Performance in Treating SWHT

The SIPP filters were cleaned again before filtration of SWHT. The reasons for cleaning were as highlighted before (significant decrease in flow rates and decline in the reduction of chemical contaminants). Although there was improved removal of some contaminants, it was hypothesised that cleaning might improve it even further. The flow rates were also low even after cleaning. It was interesting to investigate whether there would be improvement in flow rates after cleaning.

On filtration of SWHT, all contaminants (except for iron) were largely removed compared to previously determined reductions when SWLT, GWLT and GWHT samples were filtered. The contaminants (turbidity, calcium, magnesium, iron and fluorides) were significantly reduced to levels below the SANS 241 and WHO recommended guideline values. The improved reductions of the contaminants could be associated with the turbidity of the feed water. Higher reductions were observed at higher feed-water turbidity. The reductions could not be linked to filter flow rates which remained constant at about 0.5 L/h. It may also be hypothesised that filter cleaning in addition to higher turbidity of feed water resulted in improved reduction of contaminants. There was no significant difference in the hourly reduction of these contaminants during filtration of SWHT (*p* ≥ 0.05). Removal efficiency of the SIPP filters was found to be related to the concentrations of analytes in the feed water. The reduction efficiency was improved at higher initial concentrations of calcium, magnesium, iron and fluoride.

Based on the results of this study, the removal of ‘chemical’ contaminants was believed to be associated with the removal of particulate matter as well as the removal of dissolved chemicals. This finding can be supported by the observation that there was higher removal of contaminants on filtration of GWHT and SWHT (Table 3). Results have also shown much lower TOC removal by the filter than expected. There was no correlation between TOC reduction and turbidity reduction by the filter. It may be inferred that the SIPP filters were the sources of TOC due to the fact that not enough deionised water was passed through the filter before proceeding with the filtration of environmental samples. However, TOC leaching from the filters cannot be determined as it was not tested in deionised water before and after filtration when baseline flow rates were quantified. It is interesting to note that TOC reduction improved with increase in the volume of water filtered through the filter.

### 3.3. Statistical Analysis of Correlation between Removals and Initial Concentrations per Water Source

Table 4 shows statistical results of the correlation between the initial concentration of analyte in the feed water and observed removal. Results showed that the reduction of turbidity, iron and fluorides for all water sources increased with increasing concentration in the feed water.

The reverse observation was true for arsenic and total organic carbon removal for all water source samples; the highest removal of these contaminants was achieved at lower initial concentrations in the feed water. Calcium and magnesium behaved strangely. Calcium reduction was higher at higher initial concentrations on filtration of surface waters. On filtration of groundwater sources, lower initial concentrations resulted in higher observed removals. Magnesium reduction, on the other hand, was highest when the initial concentration in feed water was higher on filtration of water sources with lower turbidity (SWLT and GWLT). However, on filtration of water sources with higher turbidity (SWHT and GWHT), lower concentrations in the feed water resulted in higher removals of magnesium.

**Table 4:** Correlation between observed reductions and initial feed water concentrations.

Water Type	Relation between contaminant removal and initial concentration in source water ( *r* value)						
	Turbidity	Ca	Mg	Fe	As	F	TOC
SWLT	0.90	0.91	0.09	0.50	−0.23	0.78	−0.92
GWLT	0.68	−0.87	0.79	0.05	−0.78	0.52	−0.99
GWHT	0.24	−0.55	−0.63	0.81	−0.77	0.58	−0.99
SWHT	0.56	0.93	−0.96	0.99	−0.99	0.62	−0.68

### 3.4. Statistical Analysis of Variance in Contaminant Removal between the Water Sources

Comparison of the overall performance of the filters in removing contaminants was made. Contaminant removal efficiencies for all the water sources were compared in order to evaluate whether the filters performed similarly in removing contaminants from one source compared to the other sources. At first, all four water sources were compared. In cases where there were differences in the filters’ performances, the water source that resulted in the deviation was identified. The reduction of all analytes, with the exception of calcium, remained the same for all water sources. The probabilities (*p* values) were *p* = 0.14, *p* = 0.22, *p* = 0.65 and *p* = 0.71 for magnesium, iron, arsenic, fluoride and TOC removals respectively. Further analysis of results showed that calcium reduction on filtration of SWLT and GWHT remained the same (*p* ≥ 0.05). However, calcium reduction was higher on filtration of GWLT and SWHT compared to observed removals on filtration of SWLT and GWHT. Calcium removal on filtration of GWLT was found to be similar to removals on filtration of SWHT (*p* ≥ 0.05). 

### 3.5. Association of Chlorophyll *a* with the Flow Rate of SIPP

Results showed that higher concentrations of chlorophyll *a* were associated with lower flow rates. At higher chlorophyll *a* concentrations (70.5 mg/m^3^, for example), the turbidity of the filtered water increased (43.8 NTU) and a decrease in the flow rates of the SIPP filters (0.65 L/h) was noted. Chlorophyll *a* is an indicator of algal biomass. Although these observations were made, the correlation between flow rates and chlorophyll *a* concentrations was very weak (*r* = −0.23). It was therefore inferred that chlorophyll *a* was not the main cause of flow-rate decline due to insignificant negative correlation. It was found that chlorophyll *a* concentration was positively correlated with turbidity (*r* = 0.61); at lower chlorophyll *a* concentrations (11.2 mg/m^3^), the turbidity of the water was low (2.55 NTU). Microscopic plants which contain chlorophyll *a* added to the amount of dissolved or suspended particles in the filtered water which was measured as turbidity (NTU). 

## 4. Cost and Maintenance Guidelines of the SIPP Filter

The total manufacturing cost of a SIPP filter is between South African Rand (ZAR) 150 and ZAR 200 (USD 24 or £ 20). It has to be noted that each SIPP filter was placed in a receptacle (10 L bucket costing ZAR 15 or USD 2 or £ 2) and the receptacle was put on top of a 25 L bucket (ZAR 26 or USD 3 or £ 2) fitted with a spigot (ZAR 50 or USD 6 or £ 5). The total cost of the housing and collection system was ZAR 91 (USD 11 or £ 9). The total price for a complete SIPP filter is between ZAR 241 (USD 30 or £ 24) and ZAR 291 (USD 36 or £ 29). Before use, the insides of the SIPP filters were thoroughly scrubbed using a brush and rinsed several times with clean water. The plastic receptacles and their lids were cleaned using soapy water and rinsed several times with clean water and they were left to air-dry. The receptacles were cleaned once a week throughout the study period. It is recommended that a little household bleach (Jik) be added to the cleaning water for extra disinfection. The filter elements are made of clay and are thus fragile. They must be handled with care to avoid cracks and breakage. 

## 5. Conclusions

In conclusion, two SIPP filters were evaluated for chemical contaminant removal efficiency. The filters were able to achieve high reduction of analytes from contaminated water (Table 2). The SIPP filters also reduced turbidity by over 50% to levels allowable in terms of SANS 241 [6] as well as WHO drinking water guideline values [14]. Arsenic and fluorides were not reduced to levels allowable by SANS 241 [6] which are 10 µg/L to 50 µg/L and 1.0 mg/L to 1.5 mg/L, respectively, on filtration of all water samples for arsenic and SWLT and GWLT for fluorides. The recommended guideline values are 0.2 mg/L to 2 mg/L, 150 mg/L to 300 mg/L, 70 mg/L to 100 mg/L and 1 NTU to 5 NTU for Fe, Ca, Mg and turbidity, respectively. The SIPP filters were observed to reduce TOC and turbidity from contaminated water. Chlorophyll *a* concentrations were associated with a decrease in the flow rate of the SIPP filters. Higher chlorophyll *a* concentrations in the unfiltered water resulted in overall observation of higher turbidity in the contaminated water. Further to this present study, current work involves evaluating the SIPP filters’ life spans and evaluation of the effect of initial analyte concentration on the removal efficiency by the filters.

## 6. Study Limitations

This study has some limitations which have to be highlighted. Since only two filters were evaluated, results presented here may not be applicable to other filters due to variations in filter efficacy based on manufacturing process. The filters were only cleaned twice after filtration of GWLT and GWHT. The effects of previously retained contaminants on the filters’ performances were not quantified. It would be recommended to evaluate the filters’ performances when they are cleaned before filtration of each water source and compare results to filters which are not cleaned in between filtrations of different water sources. The filters performed poorly in reducing TOC from contaminated water but the performance improved as more water was filtered through the filters. The filters may have not been flushed enough with deionised water. This could have resulted in TOC leaching from the filters to effluent water. It is recommended that future studies investigate the volume of deionised water adequate to clean filters before filtration of contaminated water for consumption. The filters were evaluated with a total volume of 305 L for a short period; it is recommended that future studies investigate the filters’ performances over time to determine their life spans. Most importantly investigation of long term use would be helpful in quantifying the period between when arsenic is removed and when it starts flowing through the filters.
